# Bacterial and viral co-infections in patients with severe SARS-CoV-2 pneumonia admitted to a French ICU

**DOI:** 10.1186/s13613-020-00736-x

**Published:** 2020-09-07

**Authors:** Damien Contou, Aurore Claudinon, Olivier Pajot, Maïté Micaëlo, Pascale Longuet Flandre, Marie Dubert, Radj Cally, Elsa Logre, Megan Fraissé, Hervé Mentec, Gaëtan Plantefève

**Affiliations:** 1 Service de Réanimation Polyvalente, Centre Hospitalier Victor Dupouy, 69, rue du Lieutenant-Colonel Prud’hon, 95100 Argenteuil, France; 2 Service de Microbiologie, Centre Hospitalier Victor Dupouy, 69, rue du Lieutenant-Colonel Prud’hon, 95100 Argenteuil, France; 3 Equipe Mobile de Maladies Infectieuses, Centre Hospitalier Victor Dupouy, 69, rue du Lieutenant-Colonel Prud’hon, 95100 Argenteuil, France; 4 Service de Maladies Infectieuses Et Tropicales, Hôpital Bichat, Assistance Publique - Hôpitaux de Paris, 46, rue Henri Huchard, 75018 Paris, France

**Keywords:** COVID-19, SARS-CoV-2, Bacteria, Co-infection, *Staphylococcus aureus*, *Streptococcus pneumoniae*, *Haemophilus influenzae*

## Abstract

**Background:**

Data on the prevalence of bacterial and viral co-infections among patients admitted to the ICU for acute respiratory failure related to SARS-CoV-2 pneumonia are lacking. We aimed to assess the rate of bacterial and viral co-infections, as well as to report the most common micro-organisms involved in patients admitted to the ICU for severe SARS-CoV-2 pneumonia.

**Patients and methods:**

In this monocenter retrospective study, we reviewed all the respiratory microbiological investigations performed within the first 48 h of ICU admission of COVID-19 patients (RT-PCR positive for SARS-CoV-2) admitted for acute respiratory failure.

**Results:**

From March 13th to April 16th 2020, a total of 92 adult patients (median age: 61 years, 1st–3rd quartiles [55–70]; males: *n* = 73/92, 79%; baseline SOFA: 4 [3–7] and SAPS II: 31 [21–40]; invasive mechanical ventilation: *n* = 83/92, 90%; ICU mortality: *n* = 45/92, 49%) were admitted to our 40-bed ICU for acute respiratory failure due to SARS-CoV-2 pneumonia. Among them, 26 (28%) were considered as co-infected with a pathogenic bacterium at ICU admission with no co-infection related to atypical bacteria or viruses. The distribution of the 32 bacteria isolated from culture and/or respiratory PCRs was as follows: methicillin-sensitive *Staphylococcus aureus* (*n* = 10/32, 31%), *Haemophilus influenzae* (*n* = 7/32, 22%), *Streptococcus pneumoniae* (*n* = 6/32, 19%), Enterobacteriaceae (*n* = 5/32, 16%), *Pseudomonas aeruginosa* (*n* = 2/32, 6%), *Moraxella catarrhalis* (*n* = 1/32, 3%) and *Acinetobacter baumannii* (*n* = 1/32, 3%). Among the 24 pathogenic bacteria isolated from culture, 2 (8%) and 5 (21%) were resistant to 3rd generation cephalosporin and to amoxicillin–clavulanate combination, respectively.

**Conclusions:**

We report on a 28% rate of bacterial co-infection at ICU admission of patients with severe SARSCoV-2 pneumonia, mostly related to *Staphylococcus aureus, Haemophilus influenzae*, *Streptococcus pneumoniae* and Enterobacteriaceae. In French patients with confirmed severe SARSCoV-2 pneumonia requiring ICU admission, our results encourage the systematic administration of an empiric antibiotic monotherapy with a 3rd generation cephalosporin, with a prompt de-escalation as soon as possible. Further larger studies are needed to assess the real prevalence and the predictors of co-infection together with its prognostic impact on critically ill patients with severe SARS-CoV-2 pneumonia.

## Background

Severe acute respiratory syndrome coronavirus-2 (SARS-CoV-2) is the novel coronavirus originating from Wuhan, China, responsible for the illness named Coronavirus disease 2019 (COVID-19) that has rapidly spread worldwide especially in Europe. As of April 16th, 2020, the total number of French patients diagnosed with COVID-19 was 108.847, among which 6.248 were hospitalized in the intensive care unit (ICU) for acute respiratory failure. Chinese [1–3] and American [4, 5] reports on critically ill patients with COVID-19 describe a poor outcome with high mortality rate, especially in those requiring invasive mechanical ventilation. Because of the initial severity of these critically ill patients together with the complexity of ruling out an associated bacterial co-infection with clinical, biological or radiological findings [1, 6–10], more than 90% of the critically ill patients with severe SARS-CoV-2 pneumonia received an empiric antibiotic therapy upon ICU admission [1, 6, 11, 12]. However, data on the prevalence of bacterial co-infections are limited and the micro-organisms responsible for these bacterial co-infections among critically ill patients with severe SARS-CoV-2 pneumonia remain unknown [13, 14]. Similarly, there are only few data on viral co-infections [15], especially influenza co-infections, in patients with severe SARS-CoV-2 pneumonia requiring ICU admission.

We aimed to assess the rate of bacterial and viral co-infections in patients admitted to the ICU for severe SARS-CoV-2 pneumonia as well as to report the most common micro-organisms involved.

## Methods

We conducted a monocenter retrospective study including all adult (≥ 18 years old) patients admitted to our 40-bed COVID-19 ICU (Argenteuil, France) for acute respiratory failure related to SARS-CoV-2 (RT-PCR positive for SARS-CoV-2 on a nasopharyngeal swab or respiratory tract secretions) pneumonia.

All the microbiological investigations—blood culture, culture of the respiratory tract secretions, multiplex respiratory PCRs performed on a nasopharyngeal swab or on respiratory tract secretions, urinary antigen test (BinaxNOW^®^-Abbott) for *Legionella pneumophila* and *Streptococcus pneumoniae*—performed within the first 48 h of ICU admission were retrospectively reviewed. Microbiological investigations obtained more than 48 h after ICU admission were not considered in order not to include patients with nosocomial ICU-acquired pneumonia.

Three respiratory PCRs were used during the study period: a specific PCR detecting Influenza A and B (Cepheid Xpert^®^ Xpress Flu/RSV), a multiplex PCR detecting 18 bacteria and 9 viruses on respiratory tract secretions (Panel Pneumonia Plus Film array Biomerieux^®^) and a multiplex PCR detecting 4 bacteria and 15 viruses on a nasopharyngeal swab (Panel RP2 plus Film array Biomerieux^®^). These 3 PCRs are routinely used in our ICU for the diagnosis and for the treatment of patients admitted for severe lower respiratory tract infections. Given that most of the patients with SARS-CoV-2 pneumonia have no respiratory secretions with only 25–30% of them having sputum production [6, 8, 9], cultures of the respiratory tract secretions and multiplex Panel Pneumonia Plus were mostly performed in patients (under invasive mechanical ventilation or not) having respiratory tract secretions while the specific PCR detecting Influenza A and B and the multiplex PCR Panel RP2 plus were mostly performed on a nasopharyngeal swab of patients (under mechanical ventilation or not) without respiratory secretions. Laboratory exams (procalcitonin, C-reactive protein, leucocytes count) were obtained as part of the routine clinical management of patients, at the discretion of the treating intensivist in charge.

A patient was considered as co-infected when at least one of the performed microbiological investigations isolated a pathogenic bacterium (whatever the bacterial count) or a virus. The appearance of the expectorations, the values of inflammatory biomarkers and the radiological aspects were not considered to diagnose a viral or bacterial co-infection.

As recommended by the Surviving Sepsis Campaign guidelines on the management of critically ill adults with COVID-19 [16], all our patients were treated with an empiric antibiotic therapy including a third-generation cephalosporin associated with a macrolide for atypical bacteria. De-escalation was performed as soon as the results of microbiological investigations performed upon ICU admission were available. None of the patients included in the present study were treated with anti-interleukin 1 or 6, hydroxychloroquine or antiviral therapy such as remdesivir or lopinavir–ritonavir. Twenty-one patients were included (within the first 24 h of ICU admission) in a randomized controlled trial (NCT02517489) assessing the efficacity of hydrocortisone versus placebo in severe SARS-CoV-2 pneumonia [17].

Continuous variables are reported as median [interquartile range] and categorical variables are reported as numbers (percentages).

This study was conducted in accordance with the amended Declaration of Helsinki and was approved by institutional review board (IRB00011642) of the Société de Pathologie Infectieuse de Langue Française (CER-MIT 2020-0402).

## Results

From March 13th to April 16th 2020, a total of 92 adult patients were admitted to our ICU for acute respiratory failure due to SARS-CoV-2 pneumonia. Clinical characteristics, main comorbidities, biological data at ICU admission and outcomes in the ICU are detailed in Table 1. The proportion of patients who underwent the different microbiological investigations within the first 48 h of ICU admission is detailed in Tables 1 and 2. Ten of the 92 (11%) patients did not undergo any respiratory tract specimen or multiplex PCR (all of them had blood cultures, urinary antigen tests and specific PCR for Influenza A and B) and 12 (13%) did not have blood cultures at ICU admission (Table 1). Thirty-nine of the 92 patients (42%) received an antibiotic therapy before (> 12 h) ICU admission (Table 1) mostly with cefotaxime (*n* = 14/39, 36%), amoxicillin/clavulanate combination (*n*  = 13/39, 33%), amoxicillin (*n* = 6/39, 15%), piperacillin/tazobactam combination (*n* = 1/39, 3%) or others antibiotic therapies (*n* = 5/39, 13%). The median delay between hospitalization and ICU admission was 1 [0–4] day and 30 (33%) of the 92 patients were hospitalized in the wards for 48 h or more before ICU admission (Table 1).

**Table 1:** Main characteristics, comorbidities, biological data, microbiological investigations performed within the first 48 h of ICU admission and outcomes of 92 critically ill COVID-19 patients

	Critically ill patients with SARS-CoV-2 pneumonia *n* = 92
Age, years	61 [55–70]
Male, *n* (%)	73 (79)
Baseline SOFA	4 [3–7]
Baseline SAPS II	31 [21–40]
Main comorbidities, *n* (%)	
Obesity (body mass index ≥ 30 kg/m^2^)	38 (41)
Hypertension	59 (64)
Diabetes mellitus	35 (38)
Cardio-vascular diseases	9 (10)
Atrial fibrillation	3 (3)
Cerebro-vascular diseases	8 (9)
Venous thrombo-embolism	5 (5)
Chronic respiratory diseases^a^	18 (20)
Chronic renal failure	7 (8)
Immunocompromised status^b^	9 (10)
Before ICU admission	
Antibiotic therapy before (> 12 h) ICU admission, *n* (%)	39 (42)
Number of days between the first symptom and ICU admission	8.5 [7–12]
Number of days between hospitalization and ICU admission	1 [0–4]
Hospitalization in the wards for 48 h or more before ICU admission, *n* (%)	30 (33)
Biological data at ICU admission	
Leukocytes count, 10^3^/mm^3^	9.0 [6.8–12.2]
Lymphocytes count, 10^3^/mm^3^	0.8 [0.6–1.1]
Platelets count, 10^3^/mm^3^	226 [183–303]
C-reactive protein, mg/L	175 [131–232]
Procalcitonin, ng/mL	0.9 [0.3–2.2]
Fibrinogen, g/L	7.7 [6.1–8.8]
Microbiological investigations performed during the first 48 h of ICU admission	
Blood cultures	80 (87)
* Legionella pneumophila* urinary antigen test	88 (96)
* Streptococcus pneumoniae* urinary antigen test	88 (96)
Culture of respiratory tract secretions sample	67 (73)
Multiplex PCR Panel RP2 plus (nasopharyngeal swab)	26 (28)
Multiplex PCR Panel Pneumonia Plus (respiratory tract secretions)	30 (33)
Influenza A and B specific PCR	13 (14)
Culture of respiratory tract secretions sample or multiplex PCR	82 (89)
Outcomes in ICU	
Invasive mechanical ventilation	83 (90)
Prone positioning	55 (60)
Vasopressor support	57 (62)
Renal replacement therapy	22 (24)
ICU mortality	45 (49)

Continuous variables are reported as median [Interquartile range] and categorical variables are reported as numbers (percentages)

*SOFA* Sequential Organ Failure Assessment, *SAPSII* Simplified Acute Physiology Score II

^a^Including chronic obstructive pulmonary disease (*n* = 6) or/and obstructive sleep apnea (*n* = 12) or/and asthma (*n* = 4)

^b^Including chronic lymphocytic leukemia (*n* = 2), follicular or Hodgkin lymphoma (*n* = 2), liver transplantation (*n* = 1), long-term corticosteroid therapy (> 0.5 mg/kg for more than 3 months) (*n* = 3) or azathioprine (*n* = 1) administration

**Table 2:** Results of the microbiological investigations performed in 92 critically ill patients with severe SARS-CoV-2 pneumonia

	Performed among 92 critically ill patients with SARS-CoV-2 pneumonia *N*, (%)	Results	*N* (%)
Culture of respiratory tract secretions sample	67 (73%)	Sterile	24 (36%)
Sputum	16	Pathogenic bacteria^a^	19 (28%)
Tracheal aspirate	39	*Staphylococcus aureus*	6
Protected distal sampling	12	*Haemophilus influenzae*	6
		*Streptococcus pneumonia*	5
		*Escherichia coli*	2
		*Klebsiella pneumoniae*	2
		*Enterobacter cloacae*	1
		*Pseudomonas aeruginosa*	1
		*Acinetobacter baumannii*	1
		Oropharyngeal flora	24 (36%)
*Legionella pneumophila* urinary antigen test (BinaxNOW^®^—Abbott)	88 (96%)	Negative	88 (100%)
		Positive	0
*Streptococcus pneumoniae* urinary antigen test (BinaxNOW^®^—Abbott)	88 (96%)	Negative	87 (99%)
		Positive	1
Blood cultures	80 (87%)	Sterile	79 (99%)
		* Staphylococcus aureus*	1
Multiplex PCRs (Film Array Biomerieux^®^)	56 (61%)	No pathogen detected	45 (80%)
Panel RP2 plus on nasopharyngeal swab	26	Bacteria	
Panel Pneumonia Plus on respiratory tract secretions	30	*Staphylococcus aureus*	5
		*Haemophilus influenzae*	4
		*Pseudomonas aeruginosa*	2
		*Moraxella catarrhalis*	1
		*Acinetobacter baumannii*	1
		*Streptococcus pneumonia*	1
		*Enterobacter cloacae*	0
		*Escherichia coli*	0
		*Klebsiella aerogenes*	0
		*Klebsiella oxytoca*	0
		*Klebsiella pneumoniae*	0
		*Proteus* spp.	0
		*Serratia marcescens*	0
		*Streptococcus agalactiae*	0
		*Streptococcus pyogenes*	0
		*Bordetella pertussis*	0
		*Bordetella parapertussis*	0
		Atypical bacteria	
		*Chlamydophila pneumoniae*	0
		*Mycoplasma pneumoniae*	0
		*Legionella pneumophila*	0
		Viruses	
		Influenza A	0
		Influenza B	0
		Adenovirus	0
		Respiratory Syncytial virus	0
		Coronavirus HKU1, NL63, 229E, OC43	0
		MERS Coronavirus	0
		Human metapneumovirus	0
		Rhinovirus/enterovirus	0
		Parainfluenza virus 1, 2, 3 and 4	0
Influenza A and B specific PCR	13 (14%)	Negative	13 (100%)
		Positive	0 (0%)

^a^In five patients, two pathogenic bacteria were isolated in the same sample

**Fig. 1:**
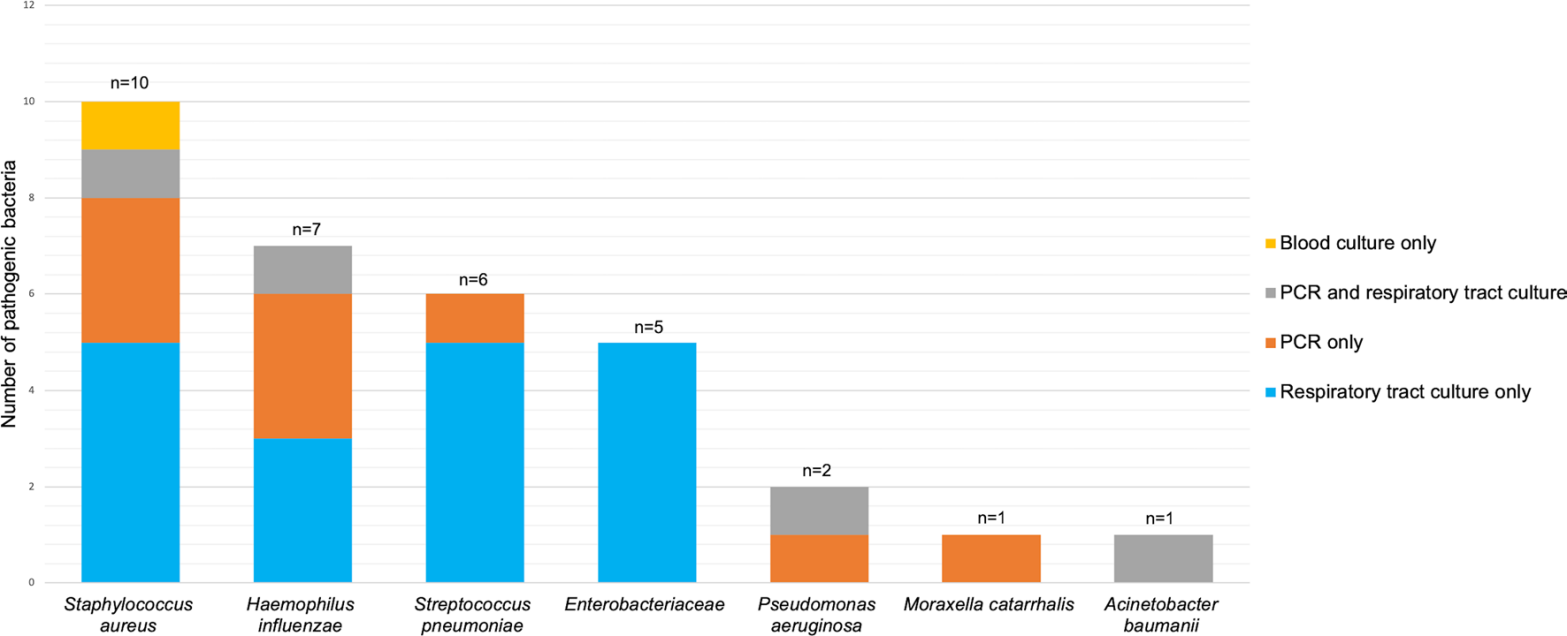
Number of each species of bacteria isolated from respiratory tract cultures (blue), multiplex PCR (red), both (grey) or blood culture (yellow) among 26 critically ill patients with severe SARS-CoV-2 pneumonia

A total of 26 (28%) patients were considered as co-infected with a pathogenic bacterium upon ICU admission while no co-infection with a virus was detected. Among the 26 co-infected patients, a total of 32 bacteria were isolated from culture and/or PCRs (Fig. 1): methicillin-sensitive *Staphylococcus aureus* (*n* = 10/32, 31%), *Haemophilus influenzae* (*n* = 7/32, 22%), *Streptococcus pneumoniae* (*n* = 6/32, 19%), Enterobacteriaceae (*n* = 5/32, 16%), *Pseudomonas aeruginosa* (*n* = 2/32, 6%), *Moraxella catarrhalis* (*n* = 1/32, 3%) and *Acinetobacter baumannii* (*n* = 1/32, 3%). When excluding the 30 patients who were hospitalized for more than 48 h before ICU admission, 18 over the remaining 62 patients (29%) were considered as having a bacterial co-infection upon ICU admission, mostly with *Staphylococcus aureus* (*n* = 5/18, 28%), *Haemophilus influenzae* (*n* = 4/18, 22%), *Streptococcus pneumoniae* (*n* = 3/18, 17%), Enterobacteriaceae (*n* = 3/18, 17%), *Pseudomonas aeruginosa* (*n* = 2/18, 11%) and *Acinetobacter baumannii* (*n* = 1/18, 6%).

Among the 67 cultures of respiratory tract secretions samples, 24 (36%) were sterile, 24 (36%) grew oropharyngeal flora and 19 (28%) isolated one (*n* = 14) or two (*n* = 5) pathogenic bacteria. The four leading micro-organisms isolated from culture of respiratory tract secretions samples were methicillin-sensitive *Staphylococcus aureus* (*n* = 6), *Haemophilus influenzae* (*n* = 6), *Streptococcus pneumoniae* (*n* = 5) and Enterobacteriaceae (*n* = 5) (Table 2). Among the 24 pathogenic bacteria isolated from culture, 2 (8%) and 5 (21%) were resistant to third-generation cephalosporin and to amoxicillin–clavulanate combination, respectively. Among the 5 patients in whom culture isolated a bacterium resistant to amoxicillin–clavulanate combination, 3 (60%) were treated with amoxicillin/clavulanate combination before ICU admission. *Legionella pneumophila* urinary antigen test was negative in 100% of the patients (*n* = 88/88) while *Streptococcus pneumoniae* urinary antigen tests was positive in only one patient (*n* = 1/88, 1%) (Table 2).

Among the 48 patients in whom cultures of the respiratory tract secretions did not isolate a pathogenic bacterium, 24 (50%) received antibiotic therapy before ICU admission. All of these 48 patients had negative urinary antigen tests for *Legionella pneumophila* and *Streptococcus pneumoniae* and all had sterile blood cultures.

Results of multiplex respiratory PCRs are detailed in Table 2. All of the 26 multiplex PCRs Panel RP2 plus (Film Array Biomerieux^®^) performed on a nasopharyngeal swab were negative. The three leading bacteria detected with the 30 multiplex PCRs Panel Pneumonia Plus (Film Array Biomerieux^®^) performed on respiratory tract secretions were methicillin-sensitive *Staphylococcus aureus* (*n* = 5), *Haemophilus influenzae* (*n* = 4) and *Pseudomonas aeruginosa* (*n* = 2). No virus was detected, especially no influenza viruses among the 68 patients tested with one of the three PCRs detecting influenza viruses.

No atypical bacterial infection was diagnosed. *Legionella pneumophila* was not detected among the 30 patients who underwent both *Legionella pneumophila* urinary antigen test and PCR for *Legionella pneumophila. Mycoplasma pneumoniae* and *Chlamydia pneumoniae* were not detected in the 56 patients who underwent PCR for *Mycoplasma pneumoniae* and *Chlamydia pneumoniae* (Table 2).

## Discussion

We herein report the first study focusing on the results of respiratory tract microbiological sampling in COVID-19 patients admitted to a French ICU for acute respiratory failure. The main results are as follows: (1) 28% of the patients admitted to our ICU for acute respiratory failure related to severe SARS-CoV-2 pneumonia might have a respiratory bacterial co-infection upon ICU admission; (2) the leading involved bacteria were methicillin-sensitive *Staphylococcus aureus*, *Streptococcus pneumoniae, Haemophilus influenzae* and Enterobacteriaceae with (3) no infection related to atypical bacteria and (4) no viral co-infection especially no influenza infection.

The prevalence of bacterial or viral co-infections in patients admitted to the ICU for acute respiratory failure related to SARS-CoV-2 pneumonia is poorly studied [13, 14]. A recent study reported on a 41% rate of co-infection among 17 patients admitted to a North American ICU [18]. A recently published analysis compiling the results of 9 studies reported on a 8% bacterial–fungal co-infection rate [12]. However, most of the included studies failed to differentiate the setting where sampling was performed (ICU versus non-ICU setting). Moreover, it is unclear whether the bacterial infections reported in these studies were community or nosocomially acquired (hospital acquired or ventilator-associated pneumonia) [12]. Last, the total number of patients undergoing microbiological sampling was poorly reported rendering difficult the calculation of a reliable rate of bacterial co-infection. In our study, we chose to focus on co-infection among the most severe patients requiring ICU admission and to exclude nosocomial ICU-acquired pneumonia.

We herein report on a 28% rate of bacterial co-infection mostly due to methicillin-sensitive *Staphylococcus aureus*, *Haemophilus influenzae, Streptococcus pneumoniae* and Enterobacteriaceae*.* The 28% rate and the bacterial spectrum observed in our cohort of severely ill COVID-19 patients are close to those reported in critically ill patients with severe seasonal [19–21] or H1N1 influenza [20, 22]*.* Noteworthy, *Pseudomonas aeruginosa* was isolated in two patients and *Acinetobacter baumannii* in one patient, none of which had risk factors such as immunosuppression, long-term corticoids therapy, chronic respiratory disease or recent hospitalization with receipt of parenteral antibiotic therapy [23].

Our 28% rate of bacterial co-infection together with the spectrum of bacteria isolated encourage the systematic administration of an empiric antibiotic therapy with a 3rd generation cephalosporin at ICU admission of patients with severe SARS-CoV-2 pneumonia (92% of the bacteria isolated from culture were susceptible to 3rd generation cephalosporins) with a prompt de-escalation as soon as the results of bacterial cultures and respiratory PCRs are available. No atypical bacteria could be detected in this cohort, which is in line with other reports [12, 15]. This questions the systematic use of antimicrobials targeting atypical bacteria in these patients. This is particularly relevant since some of these drugs have been associated with acute cardiotoxicity (QT interval prolongation and torsade de pointes) [19] a fortiori when co-administered with other drugs such as lopinavir/ritonavir or hydroxychloroquine [24, 25].

No viral co-infection was detected in our cohort of critically ill COVID-19 patients, especially no influenza viruses despite the screening of 75% of the patients included in our cohort and the active seasonal period. Our findings are conflicting with those of Kim et al. who reported on a 21% (*n* = 24/116) rate of viral co-infections with a non-SARS-CoV-2 respiratory pathogen, mostly rhinovirus/enterovirus, respiratory syncytial virus and non-SARS-CoV-2 Coronaviridae. Nevertheless, our findings combined with those of Kim et al. who reported only 1 positive patient for influenza virus among 116 patients tested, discourage the systematic prescription of an empirical antiviral treatment with neuraminidase inhibitors in critically ill patients with a confirmed SARS-CoV-2 pneumonia.

Our study has several limitations. First, the retrospective monocenter design with inherently associated bias may limit its generalizability to other centers with a different bacterial ecology. Second, half of the patients in whom cultures of the respiratory tract did not isolate a pathogenic bacterium received an antibiotic therapy prior to ICU admission, which could have influenced bacterial co-infection identification and potentially underestimated the real rate of bacterial co-infection. Third, 10 patients did not undergo respiratory tract specimen or multiplex PCR (all of them had blood cultures, urinary antigen tests and specific PCR for Influenza A and B) and 12 did not have blood cultures at ICU admission which might also participate to a potential underestimation of the real rate of co-infection. Fourth, we did not consider bacterial count which could have helped to distinguish between an infection and a respiratory tract colonization. However, only six of the 92 patients had a chronic obstructive pulmonary disease downplaying the risk of prior bronchial colonization. Moreover, since 42% of the patients received an antibiotic therapy before ICU admission, bacterial count may have been below the threshold despite the presence of an authentic bacterial co-infection. Fifth, the multiplex PCRs were mostly performed on the upper respiratory tract secretions, which might have limited their sensibility to detect viruses compared to broncho-alveolar lavage [26]. Last, we did not analyze the radiological aspects of the 92 patients which could have been of interest to determine whether the presence of alveolar condensations associated with the typical ground glass COVID-19 opacities was associated with bacterial co-infection.

Further larger studies are needed to assess the real prevalence and the predictors of co-infection together with its prognostic impact on critically ill patients with severe SARS-CoV-2 pneumonia [13]. Streamlining antibiotic stewardship is of utmost importance in COVID-19 patients. Selection pressure should be kept as low as possible in these patients who commonly experience prolonged durations of mechanical ventilation [27, 28] increasing the risk of hospital and ventilator acquired pneumonia.

## Conclusions

We report on a 28% rate of bacterial co-infection at ICU admission of patients with severe SARSCoV-2 pneumonia, mostly with *Staphylococcus aureus, Haemophilus influenzae*, *Streptococcus pneumoniae* and Enterobacteriaceae. In French patients with a confirmed severe SARSCoV-2 pneumonia requiring ICU admission, our results encourage the systematic administration of an empiric antibiotic monotherapy with a 3rd generation cephalosporin, with a prompt de-escalation as soon as possible.

## Data Availability

The dataset used and analyzed for the current study is available from the corresponding author on reasonable request.

## Abbreviations

ARDS: Acute respiratory disease syndrome
COVID-19: Coronavirus disease 2019
ICU: Intensive care unit
SARS-CoV-2: Severe acute respiratory syndrome coronavirus-2
